# Development and validation of nursing-oriented risk prediction models for anxiety and depression in hospitalized patients with chronic kidney disease: a retrospective cross-sectional study in Southwest China

**DOI:** 10.3389/fpsyt.2025.1683467

**Published:** 2026-01-15

**Authors:** Hong Xiao, Yan Xiao, Chongzhi Yin, Xin Yang, Zhaolan Yu

**Affiliations:** 1 Department of Nephrology, The Affiliated Hospital, Southwest Medical University, Luzhou, Sichuan, China; 2 Department of Oncology, The Affiliated Hospital, Southwest Medical University, Luzhou, Sichuan, China

**Keywords:** chronic kidney disease, nursing care, prediction model, psychological risk, Southwest China

## Abstract

**Background:**

Psychological disorders such as anxiety and depression are common but often underrecognized among patients with chronic kidney disease (CKD), posing challenges for inpatient nursing care. This study aimed to identify key risk factors and develop predictive models to assist clinical nurses in early psychological risk identification and intervention planning.

**Methods:**

This retrospective cross-sectional study for model development included 1,420 adult inpatients with CKD stages 1–5 admitted to a tertiary hospital in Southwest China from March 2023 to March 2025. Anxiety and depression were assessed using the Generalized Anxiety Disorder 7-item scale (GAD-7) and Patient Health Questionnaire 9-item scale (PHQ-9) within 48 hours of admission. Nursing-relevant demographic, clinical, and psychosocial data were extracted from electronic health records. Multivariate logistic regression was used to identify predictors. Two nomograms were developed, and model performance was assessed via ROC curves, calibration plots, and 1,000 bootstrap validations.

**Results:**

Screening-positive anxiety and depressive symptoms (GAD-7/PHQ-9 ≥5) were observed in 33.2% and 35.8% of patients, respectively. Significant predictors of anxiety included younger age, female sex, low income, frequent hospitalizations, hypoalbuminemia, sleep disturbance, diabetes, and absence of family accompaniment. Similar predictors were found for depression, along with low education and dialysis status. Both models demonstrated strong discrimination (AUC = 0.830 for anxiety; 0.829 for depression) and good calibration. The nomograms allow bedside nurses to estimate psychological risk using routinely available data.

**Conclusions:**

Anxiety and depression are highly prevalent among hospitalized CKD patients in Southwest China and are associated with modifiable psychosocial and clinical factors. The validated nursing-oriented prediction models offer practical tools to support early risk stratification and targeted psychological care planning in nephrology nursing practice.

## Introduction

Chronic kidney disease (CKD) is a major and growing global public health concern, affecting over 10% of the adult population worldwide (more than 800 million individuals) and contributing substantially to cardiovascular disease, hospitalization, and premature death (1). While the clinical management of CKD has advanced in recent years, psychological distress—particularly anxiety and depression—remains highly prevalent and under-recognized among this population. Recent large−scale studies have reported that approximately 30–40% of individuals with chronic kidney disease (CKD) exhibit clinically significant symptoms of anxiety or depression, with the presence of these psychological symptoms linked to poorer treatment adherence, diminished quality of life, and adverse clinical outcomes (2, 3).

The mental health needs of hospitalized CKD patients are especially critical but frequently overlooked. Hospitalization, with its associated stress, loss of autonomy, and disruption of daily routines, may further aggravate emotional distress in vulnerable individuals (4, 5). Despite these risks, mental health screening is not routinely integrated into inpatient care protocols, particular rly in nephrology wards. Nurses, as frontline healthcare providers at the bedside, are uniquely positioned to detect early psychological distress in CKD patients through continuous patient interaction and routine supportive assessments (6). Nursing records often contain rich data on sleep disturbance, family support, nutritional status, and prior hospitalization—factors that may reflect psychosocial vulnerability but are rarely leveraged in psychological risk assessment models.

While several studies have proposed prediction models for depression in CKD outpatients or dialysis populations, these models typically rely on complex or non-routine data, and most exclude in-hospital assessments (7, 8). Moreover, few models address anxiety, despite its distinct clinical relevance and frequent co-occurrence with depression (9). Importantly, prior models have rarely adopted a nursing-oriented perspective that emphasizes variables accessible during routine nursing assessments. As healthcare systems move toward precision nursing and mental health integration, developing pragmatic tools to support early, bedside identification of high-risk patients is increasingly necessary (10).

A recent cross-sectional analysis of 4,414 CKD patients from the National Health and Nutrition Examination Survey (NHANES) 2005–2018 dataset developed a nomogram incorporating sociodemographic, comorbid, and lifestyle variables to predict depression risk, achieving robust discrimination with an AUC of 0.785 (95% CI: 0.761–0.809) in the training cohort and 0.773 (95% CI: 0.737–0.810) on validation (11).

However, predictive models developed in outpatient CKD cohorts often inadequately address complexities of the inpatient environment, where acute physiological stressors and care interactions substantially differ from outpatient care (12). Similarly, depression prediction models designed for maintenance hemodialysis patients frequently depend on biochemical metrics and dialysis vintage, limiting their generalizability to broader CKD or inpatient populations (13). There remains a notable gap in predictive tools tailored to hospitalized CKD patients, especially those that draw upon nursing-accessible variables.

Therefore, the objective of this study was to develop and internally validate two predictive models—one for anxiety and one for depression—using routine demographic, clinical, and psychosocial data collected during inpatient nursing assessments. In particular, nurse-assessed variables—such as sleep disturbance and family accompaniment—represent unique, context-specific indicators of psychosocial vulnerability during hospitalization and are not available in community- or population-based datasets. These features reflect real-time patient experiences and are highly actionable within nursing workflows, underscoring the added value of a nursing-oriented predictive approach. By constructing user-friendly nomograms based on data available at the bedside, we aimed to provide practical tools for frontline nephrology nurses to identify patients at psychological risk and guide timely, targeted interventions. To our knowledge, this is the first large-scale study to develop nursing-integrated psychological risk models in a hospitalized CKD population in Southwest China.

## Methods

### Study design and setting

This was a retrospective cross-sectional study for predictive model development conducted at a large tertiary general hospital located in Southwest China. All predictor and outcome variables were measured within 48 hours of admission, and no longitudinal follow-up of psychological outcomes was performed. The study included adult patients who were consecutively admitted to the Department of Nephrology between March 1, 2023, and March 1, 2025. Clinical, demographic, and psychosocial data were obtained from the hospital’s electronic health record (EHR) system. The study aimed to examine the prevalence and determinants of anxiety and depression among hospitalized patients with chronic kidney disease (CKD), with the goal of developing predictive models to support early identification and targeted intervention. All predictors and psychological outcomes were obtained within the first 48 hours of admission, and the study therefore represents a retrospective cross-sectional model-development analysis rather than a longitudinal predictive design.

### Participants

Patients were eligible for inclusion if they met all of the following criteria: (1) age ≥18 years at the time of hospital admission; (2) a confirmed diagnosis of chronic kidney disease (CKD) stages 1 to 5 based on the Kidney Disease: Improving Global Outcomes (KDIGO) 2012 clinical practice guidelines, defined by either estimated glomerular filtration rate (eGFR) <60 mL/min/1.73 m² for ≥3 months or evidence of kidney damage (e.g., albuminuria, abnormal renal imaging, or histological abnormalities); and (3) completed standardized psychological assessments (GAD-7 and PHQ-9) within 48 hours of hospitalization as part of routine nursing evaluation.

Patients were excluded based on the following predefined criteria: (1) a prior documented diagnosis of psychiatric disorders (e.g., major depressive disorder, anxiety disorder, bipolar disorder, schizophrenia) in the EHR or referral records; (2) cognitive impairment, severe language barrier, or consciousness disturbance during assessment, as determined by attending nurses or physicians, which could compromise the validity of self-reported questionnaires; (3) incomplete or missing GAD-7 or PHQ-9 data; (4) length of hospital stay <48 hours, preventing completion of psychosocial evaluation; or (5) inter-hospital transfers or referrals from other institutions, to ensure consistency in care processes and documentation quality.

To minimize selection bias, a consecutive sampling strategy was used. After applying the inclusion and exclusion criteria, a total of 1,420 unique patients were identified and included in the final analytic cohort. Baseline characteristics and data completeness were assessed to ensure representativeness and reliability of the cohort.

### Psychological assessment

Anxiety and depression symptoms were assessed using the Chinese versions of the Generalized Anxiety Disorder 7-item scale (GAD-7) and the Patient Health Questionnaire 9-item scale (PHQ-9), respectively. Both instruments have been validated in Chinese clinical populations. Patients completed these assessments within 48 hours of admission under nurse supervision, and responses were recorded in the EHR. A score ≥5 on each scale was used to define the presence of at least mild anxiety or depressive symptoms, consistent with commonly used thresholds in validation studies; in this study, these cut-offs were intended to flag patients at increased risk of psychological distress rather than to establish a formal psychiatric diagnosis.

### Data collection and variable definition

A structured data abstraction form was used to collect variables across four domains: demographic (age, sex, education, marital status, income), clinical (CKD stage, comorbidities, serum albumin, hemoglobin), hospitalization-related (length of stay, prior hospitalizations, family accompaniment), and psychosocial (sleep disturbance). In addition, length of hospital stay was extracted for descriptive and exploratory analyses only and was not considered as a candidate predictor, because it is not available at the time of early psychological risk assessment. Two trained investigators independently extracted all data; discrepancies were resolved by discussion with a third reviewer. Inter-rater reliability was evaluated on a 10% random sample (Cohen’s κ = 0.91).

Sleep disturbance was defined based on nursing documentation using standardized EHR templates. Other symptom-related items such as appetite, general mental state, and detailed mood descriptors were not included as candidate predictors because, in our institution, they are primarily recorded as free-text narrative notes or incompletely checked forms, resulting in substantial variability and high rates of missing data. Similarly, inflammatory biomarkers (e.g., C-reactive protein, interleukin-6) are not routinely obtained for all CKD inpatients at admission and therefore did not meet our predefined data-quality threshold. In contrast, sleep disturbance represented a standardized, structured nursing assessment item with high completeness and was therefore selected as the primary symptom-level predictor. Serum albumin was dichotomized at 35 g/L per nutritional risk thresholds. Monthly income was categorized as <3,000 or ≥3,000 RMB based on local economic standards. All variable cutoffs were established *a priori* based on clinical relevance or literature support.

### Missing data management

Variables with more than 5% missing values were excluded. This criterion led to the exclusion of several potentially relevant but inconsistently recorded variables, including detailed appetite ratings, narrative assessments of general mental state, and non-routinely ordered inflammatory biomarkers. For remaining variables, missingness was assessed using Little’s MCAR test (P > 0.05), suggesting data were missing completely at random. Pairwise deletion was applied to preserve maximum information.

### Statistical analysis

Continuous variables were expressed as mean ± standard deviation (SD) or median with interquartile range (IQR), depending on distribution, and compared using Student’s t test or Mann–Whitney U test. Categorical variables were summarized as frequencies and percentages, and compared using chi-square or Fisher’s exact tests. Univariate analyses were conducted to identify candidate variables for multivariate logistic regression; length of hospital stay was examined only descriptively and in unadjusted comparisons and was not entered into the multivariable models or nomograms. In addition, variable selection was guided by a prespecified nursing-oriented conceptual framework emphasizing socioeconomic vulnerability, nutritional status, sleep disturbance, and family support, which are routinely assessed by nurses and represent theoretically grounded predictors of psychological distress in CKD inpatients.

Multicollinearity among predictors was assessed using variance inflation factors (VIFs), all of which were <2. Backward stepwise logistic regression models were constructed separately for anxiety and depression, with entry and removal criteria set at P < 0.05. Adjusted odds ratios (aORs) and 95% confidence intervals (CIs) were reported.

### Model evaluation

Model discrimination was assessed by calculating the area under the receiver operating characteristic curve (AUC) with 95% CIs. Optimal cut-off values were determined using the maximum Youden Index. Calibration was evaluated using the Hosmer-Lemeshow goodness-of-fit test and calibration plots. Internal validation was performed using 1,000 bootstrap replications that repeated the full modeling process, including variable selection, to assess optimism-corrected performance. This internal bootstrap process provides optimism-corrected estimates of model performance but does not replace external validation. Nomograms were constructed for both models to facilitate bedside clinical application. In addition, we derived the corresponding logistic regression equations and implemented two R Shiny–based risk calculators; the full equations (intercepts and β-coefficients for all predictors), the annotated R code, and screenshots of the user interface are provided as **Supplementary Material 1** to support point-of-care use and independent replication. Because this study was designed as a model-development cross-sectional analysis, the entire dataset was used for model construction, and bootstrap resampling served exclusively as an internal validation strategy rather than a substitute for external validation. To further enhance model robustness, LASSO regularization was applied prior to multivariable modeling to identify stable predictors and reduce overfitting.

For both anxiety and depression, the prediction models follow the standard logistic regression form, logit(P) = β_0_ + Σβ_i_X_i_, where X_i_ denotes the presence (1) or absence (0) of each risk factor and β_i_ represents the corresponding regression coefficient. The β-coefficients are the natural logarithms of the adjusted odds ratios listed in **Tables 1**, **2**, and the model intercepts were obtained from the multivariable regression output. To facilitate bedside use, **Supplementary Material 1** includes (i) the complete model equations with all β-coefficients and intercepts, (ii) a step-by-step worked example demonstrating how to calculate an individual patient’s predicted probability, and (iii) the R Shiny code for the web-based calculators, which automatically compute the probability of anxiety or depression based on the eight or nine predictors. All analyses were performed using SPSS version 26.0 (IBM Corp., Armonk, NY) and R version 4.2.2 (R Foundation for Statistical Computing, Vienna, Austria). A two-sided P value < 0.05 was considered statistically significant.

**Table 1:** Multivariate logistic regression analysis identifying independent predictors of anxiety in hospitalized patients with chronic kidney disease (N = 1,420).

Variable	aOR (95% CI)	P value
Age ≥ 60 years (Ref: <60)	0.73 (0.57–0.94)	0.013
Female sex (Ref: Male)	1.34 (1.02–1.76)	0.034
Monthly income <3,000 RMB (Ref: ≥3,000)	1.51 (1.17–1.97)	0.002
≥2 hospitalizations in past year (Ref: <2)	1.59 (1.20–2.11)	0.001
Serum albumin <35 g/L (Ref: ≥35 g/L)	1.58 (1.22–2.06)	<0.001
Sleep disturbance (Ref: No)	2.34 (1.82–3.01)	<0.001
No family accompaniment (Ref: Accompanied)	1.66 (1.22–2.26)	0.001
Diabetes mellitus (Ref: No)	1.28 (1.00–1.64)	0.047

aOR, adjusted odds ratio; CI, confidence interval; CKD, chronic kidney disease; RMB, renminbi (Chinese yuan); g/L, grams per liter. Anxiety was defined as a Generalized Anxiety Disorder 7-item (GAD-7) score ≥5. Candidate variables were first screened using univariate analyses and then further evaluated through 10-fold cross-validated least absolute shrinkage and selection operator (LASSO) regression to minimize overfitting and reduce data-driven bias. Variables with non-zero LASSO coefficients, combined with established clinical relevance, were subsequently entered into the multivariate logistic regression models. Multicollinearity was assessed using variance inflation factors (VIFs), all <2. Model calibration was evaluated with the Hosmer-Lemeshow goodness-of-fit test (P = 0.41), and explanatory power assessed using Nagelkerke R² = 0.26. All variables were clinically defined, and cutoff values (e.g., age ≥60 years, albumin <35 g/L) were based on established guidelines or relevant clinical thresholds.

**Table 2:** Multivariate logistic regression analysis of factors associated with depression in hospitalized patients with chronic kidney disease (N = 1,420).

Variable	aOR (95% CI)	P value
Age ≥ 60 years (Ref: <60)	0.75 (0.59–0.96)	0.022
Female sex (Ref: Male)	1.38 (1.06–1.79)	0.015
Low education (≤ middle school) (Ref: > middle school)	1.42 (1.09–1.84)	0.008
Monthly income <3,000 RMB (Ref: ≥3,000)	1.58 (1.23–2.04)	<0.001
CKD stage 5 on dialysis (Ref: Stage 1–4)	1.47 (1.10–1.96)	0.009
≥2 hospitalizations in past year (Ref: <2)	1.63 (1.24–2.14)	<0.001
Serum albumin <35 g/L (Ref: ≥35 g/L)	1.49 (1.14–1.94)	0.003
Sleep disturbance (Ref: No)	2.63 (2.04–3.39)	<0.001
No family accompaniment (Ref: Accompanied)	1.59 (1.19–2.12)	0.002

aOR, adjusted odds ratio; CI, confidence interval; CKD, chronic kidney disease; RMB, renminbi (Chinese yuan); g/L, grams per liter. Depression was defined as a Patient Health Questionnaire-9 (PHQ-9) score ≥5. All independent variables with P < 0.05 in univariate comparisons were included in a backward stepwise logistic regression model. Education level and marital status were retained based on both univariate significance and theoretical relevance. Reference categories are listed in parentheses. Multicollinearity was assessed using variance inflation factors (VIFs), all <2. Model calibration was acceptable (Hosmer–Lemeshow test P = 0.44), and overall explanatory power was moderate (Nagelkerke R² = 0.29). Variables such as sleep disturbance, nutritional status, frequency of hospitalization, and family support represent modifiable factors, which may be targeted by nursing interventions.

## Results

### Baseline characteristics

A total of 1,420 hospitalized patients with chronic kidney disease (CKD) were included in the analysis. The mean age was 61.4 years (SD 13.8), and 42.0% of the patients were female. More than half of the participants (58.0%) had an education level of middle school or below, and 39.9% reported a monthly income of less than 3,000 RMB. Regarding disease stage, 14.6% of patients were undergoing dialysis. The prevalence of key comorbidities was high, including hypertension (74.1%), diabetes mellitus (51.7%), and anemia (62.8%). Notably, 44.8% of patients reported sleep disturbance, and 17.4% were hospitalized without family accompaniment (**Table 3**). Length of hospital stay is presented for descriptive purposes and was not used as a predictor in model development.

**Table 3:** Baseline characteristics of hospitalized patients with chronic kidney disease (N = 1,420).

Variable	Value
Age, years, mean ± SD	61.4 ± 13.8
Sex, n (%)	
Male	824 (58.0)
Female	596 (42.0)
Education level, n (%)	
≤ Middle school	823 (58.0)
> Middle school	597 (42.0)
Marital status, n (%)	
Married/cohabiting	1,128 (79.4)
Unmarried/divorced/widowed	292 (20.6)
Monthly income (RMB), n (%)	
<3,000	566 (39.9)
≥3,000	854 (60.1)
CKD stage, n (%)	
Stage 1–3	556 (39.2)
Stage 4–5 (non-dialysis)	656 (46.2)
Stage 5 (dialysis)	208 (14.6)
Comorbidities, n (%)	
Hypertension	1,052 (74.1)
Diabetes mellitus	734 (51.7)
Cardiovascular disease	385 (27.1)
Anemia	892 (62.8)
Hospital stay, days, median [IQR]	9 [6–13]
Previous hospitalizations in past year, n (%)	
0	596 (42.0)
1	498 (35.1)
≥2	326 (22.9)
Serum albumin, g/L, mean ± SD	35.2 ± 5.7
Sleep disturbance reported, n (%)	636 (44.8)
Family accompaniment during hospitalization, n (%)	1,173 (82.6)

CKD, chronic kidney disease; SD, standard deviation; IQR, interquartile range; RMB, renminbi (Chinese yuan); g/L, grams per liter. Sleep disturbance was extracted from nursing documentation. No variables had more than 5% missing data; missing data were handled with pairwise deletion.

### Factors associated with anxiety

Among the 1,420 patients, 472 (33.2%) screened positive for anxiety symptoms based on a GAD-7 score ≥5. Compared to patients without anxiety, those with anxiety were younger, more likely to be female, have lower education and income, more frequent hospitalizations, and lower serum albumin levels. They also had significantly higher rates of anemia, diabetes, cardiovascular disease, sleep disturbance, and hospitalization without family support (all P < 0.05; **Table 4**). In the multivariate logistic regression analysis, eight variables emerged as independent predictors of anxiety. Protective effects were observed for older age (aOR = 0.73, 95% CI: 0.57–0.94, P = 0.013), while increased risk was associated with female sex (aOR = 1.34), low income (aOR = 1.51), frequent hospitalizations (aOR = 1.59), low serum albumin (aOR = 1.58), sleep disturbance (aOR = 2.34), lack of family accompaniment (aOR = 1.66), and diabetes mellitus (aOR = 1.28) (all P < 0.05; **Table 1**). LASSO regression identified the same eight predictors with non-zero coefficients, supporting the robustness of variable selection (**Supplementary Table 1**, **Supplementary Figure S1**).

**Table 4:** Comparison of key characteristics between patients with and without anxiety (N = 1,420).

Variable	Anxiety (n = 472)	No anxiety (n = 948)	P value
Age, years, mean ± SD	59.1 ± 13.2	62.6 ± 14.0	<0.001
Female sex, n (%)	220 (46.6)	376 (39.7)	0.018
Lower education (≤ middle school), n (%)	295 (62.5)	528 (55.7)	0.017
Unmarried/divorced/widowed, n (%)	113 (23.9)	179 (18.9)	0.024
Low income (<3,000 RMB/month), n (%)	232 (49.2)	334 (35.2)	<0.001
CKD stage 5 on dialysis, n (%)	81 (17.2)	127 (13.4)	0.046
≥2 hospitalizations in past year, n (%)	139 (29.4)	187 (19.7)	<0.001
Serum albumin, g/L, mean ± SD	33.8 ± 5.5	36.0 ± 5.6	<0.001
Anemia, n (%)	324 (68.6)	568 (59.9)	0.002
Diabetes mellitus, n (%)	268 (56.8)	466 (49.2)	0.01
Cardiovascular disease, n (%)	145 (30.7)	240 (25.3)	0.041
Sleep disturbance, n (%)	298 (63.1)	338 (35.6)	<0.001
No family accompaniment during hospitalization, n (%)	111 (23.5)	136 (14.4)	<0.001
Length of stay, days, median [IQR]	10 [7–14]	9 [6–12]	0.007

CKD, chronic kidney disease; SD, standard deviation; IQR, interquartile range; RMB, renminbi (Chinese yuan); g/L, grams per liter. Anxiety was defined as a Generalized Anxiety Disorder 7-item (GAD-7) score ≥5. Categorical variables were compared using the chi-square test, continuous variables were analyzed using independent-samples t test or Mann–Whitney U test depending on data distribution. This table presents unadjusted comparisons to identify candidate variables for multivariate logistic regression analysis; statistically significant results (P < 0.05) suggest potential associations but not causation. Length of hospital stay was evaluated only in these unadjusted comparisons and was not included as a predictor in the final multivariable models.

### Factors associated with depression

Similarly, 509 patients (35.8%) screened positive for depressive symptoms (PHQ-9 ≥5). In the adjusted model, depression was significantly associated with female sex (aOR = 1.38, 95% CI: 1.06–1.79), low education (aOR = 1.42), low income (aOR = 1.58), dialysis status (aOR = 1.47), frequent hospitalizations (aOR = 1.63), low albumin levels (aOR = 1.49), sleep disturbance (aOR = 2.63), and absence of family accompaniment (aOR = 1.59). Age ≥60 years remained a protective factor (aOR = 0.75, P = 0.022; **Table 2**). LASSO regression identified the same eight predictors with non-zero coefficients, supporting the robustness of variable selection (**Supplementary Table 1**, **Supplementary Figure S1**).

### Predictive performance of the models

**Figure 1:**
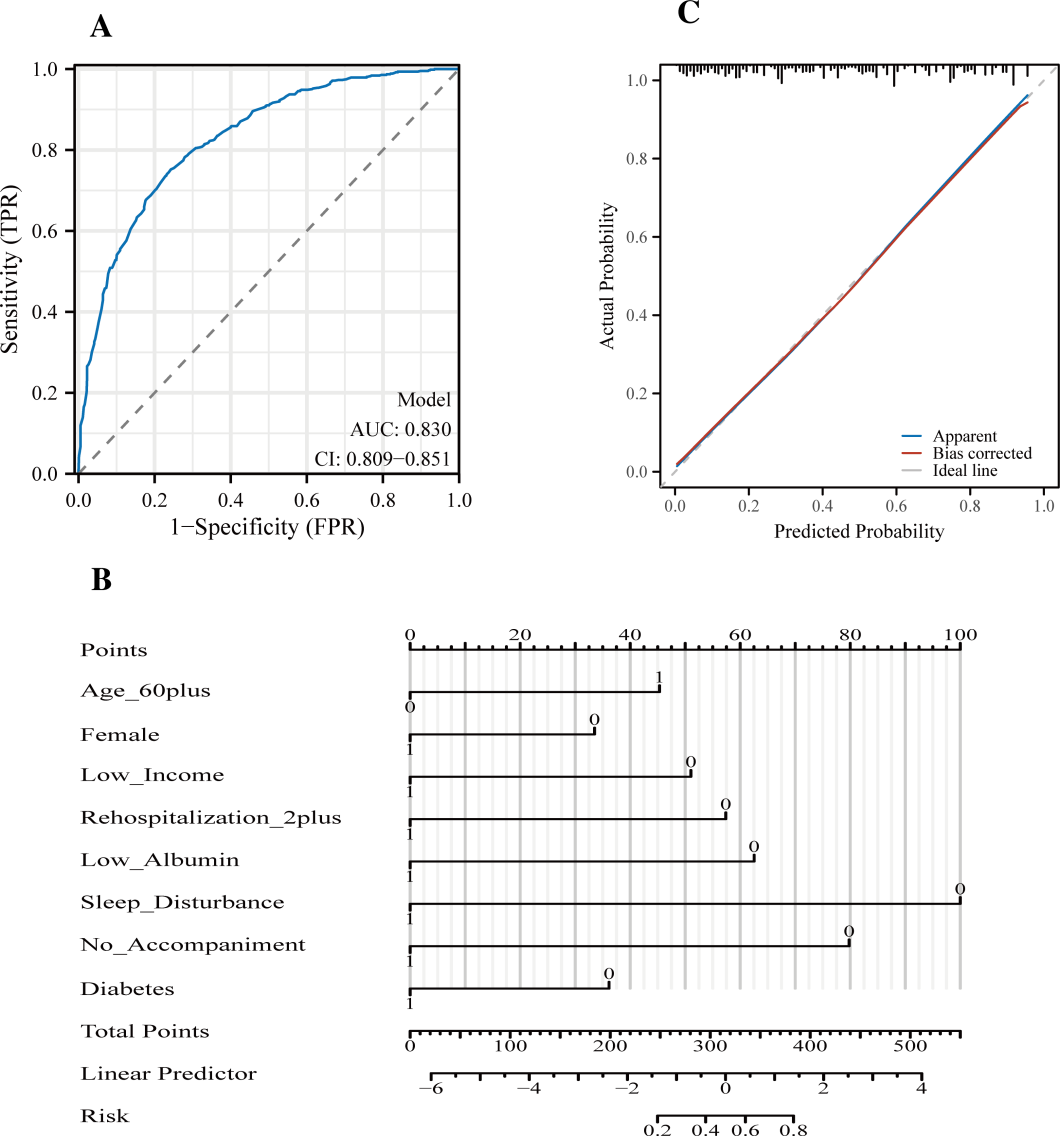
Performance of the nursing-oriented predictive model for anxiety in hospitalized patients with chronic kidney disease (CKD). **(A)** Receiver operating characteristic (ROC) curve of the anxiety prediction model, demonstrating excellent discriminative ability with an area under the curve (AUC) of 0.830 (95% confidence interval [CI]: 0.809–0.851). **(B)** Nomogram for estimating the probability of anxiety at hospital admission based on eight independent predictors: age ≥60 years, female sex, low income, ≥2 prior hospitalizations, hypoalbuminemia, sleep disturbance, absence of family accompaniment, and diabetes mellitus. Each predictor corresponds to a point value; total points are summed and mapped to the predicted risk at the bottom scale. **(C)** Calibration plot of the anxiety prediction model, showing good agreement between predicted and observed probabilities in the derivation cohort. The apparent curve, bias-corrected curve (bootstrap resampling, n = 1,000), and ideal reference line are displayed. The underlying logistic regression equation and the R Shiny code for the anxiety risk calculator are provided in **Supplementary Material 1**.

The multivariate logistic regression models for anxiety and depression exhibited strong predictive performance in hospitalized patients with chronic kidney disease. For the anxiety model, the area under the receiver operating characteristic (ROC) curve was 0.830 (95% CI: 0.809–0.851), indicating excellent discriminative ability (**Figure 1A**). At the optimal cutoff based on the Youden Index, the model achieved a sensitivity of 75.2% and specificity of 75.7%, with positive and negative predictive values of 70.9% and 79.5%, respectively (**Table 5**). The corresponding nomogram (**Figure 1B**) allows for individualized probability estimation based on the weighted contributions of each risk factor. The calibration curve (**Figure 1C**) further demonstrated good agreement between predicted and observed probabilities, with minimal deviation from the ideal reference line.

**Table 5:** Predictive performance of multivariate logistic regression models for anxiety and depression in hospitalized patients with chronic kidney disease.

Outcome	AUC (95% CI)	Sensitivity	Specificity	PPV	NPV
Anxiety (GAD-7 ≥ 5)	0.830 (0.809–0.851)	75.20%	75.70%	70.90%	79.50%
Depression (PHQ-9 ≥ 5)	0.829 (0.807–0.850)	74.20%	78.10%	78.00%	74.30%

AUC, area under the receiver operating characteristic curve; CI, confidence interval; PPV, positive predictive value; NPV, negative predictive value; GAD-7, Generalized Anxiety Disorder 7-item scale; PHQ-9, Patient Health Questionnaire 9-item depression scale. Predictive performance of the logistic regression models was assessed using the same dataset used for model construction. Sensitivity, specificity, PPV, and NPV were calculated at the optimal cutoff point determined by the maximum Youden Index. The anxiety prediction model demonstrated excellent discriminative ability, with an AUC of 0.830, indicating strong potential for clinical application in early identification and targeted nursing intervention.

**Figure 2:**
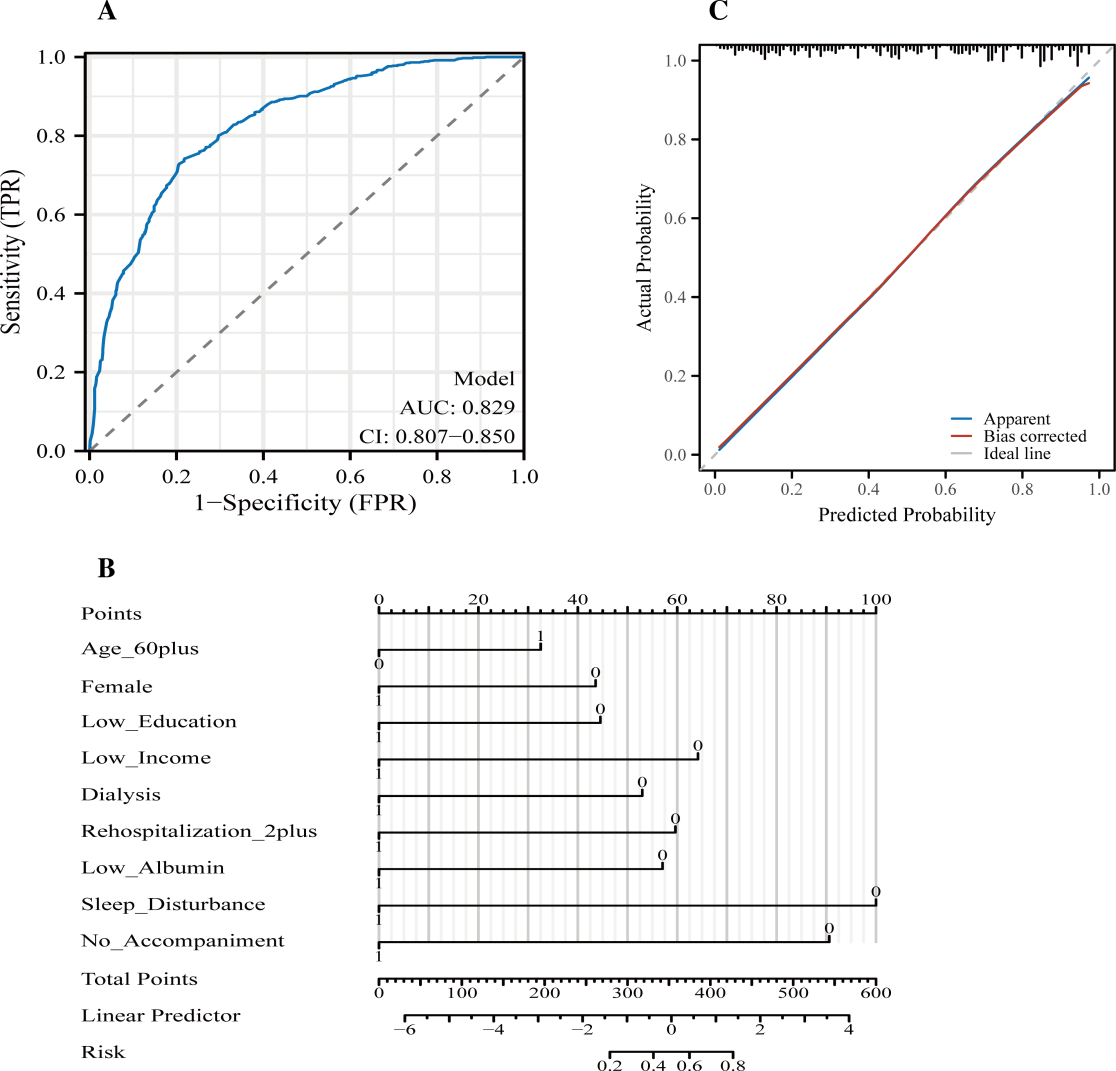
Performance of the depression prediction model in hospitalized patients with chronic kidney disease (CKD). **(A)** Receiver operating characteristic (ROC) curve of the depression prediction model, demonstrating excellent discriminative performance with an area under the curve (AUC) of 0.829 (95% confidence interval [CI]: 0.807–0.850). **(B)** Nomogram for estimating the probability of depression at hospital admission. Each predictor corresponds to a point value, and the sum of all points is mapped to the linear predictor and the final estimated risk at the bottom scale. Predictors incorporated in the model include: age ≥60 years, female sex, low education (≤ middle school), low income (<3,000 RMB), dialysis status, ≥2 hospitalizations in the previous year, serum albumin <35 g/L, sleep disturbance, and no family accompaniment. **(C)** Calibration plot of the depression model showing good agreement between predicted and observed risk. The apparent curve and the bootstrap bias-corrected curve (1,000 repetitions) closely follow the ideal diagonal reference line, indicating satisfactory model calibration across the entire probability range.

Similarly, the depression prediction model also performed well, yielding an AUC of 0.829 (95% CI: 0.807–0.850) (**Figure 2A**). The model showed a sensitivity of 74.2%, specificity of 78.1%, PPV of 78.0%, and NPV of 74.3%. A visual nomogram (**Figure 2B**) was constructed to facilitate clinical interpretation and bedside application, while the calibration plot (**Figure 2C**) confirmed the model’s accuracy in probability estimation across the entire risk spectrum. Collectively, these findings support the practical utility of both models in early psychological risk stratification and targeted nursing interventions in CKD inpatient settings. For illustration, we also provide a worked example using the depression risk calculator included in **Supplementary Material 1**. When all predictors are set to “No” (Age ≥60 = 0, Female = 0, Low education = 0, Low income = 0, Dialysis patient = 0, ≥2 hospitalizations = 0, Albumin <35 g/L = 0, Sleep disturbance = 0, and No family accompaniment = 0), the calculator yields a predicted probability of depression of approximately 0.36 (36%). This example demonstrates the transparency of the model and the ease with which nurses can obtain individualized risk estimates at the bedside.

### Decision-curve analysis

To evaluate clinical usefulness, we performed a decision-curve analysis (DCA) for both models. The anxiety model demonstrated a higher net benefit than the “treat-all” and “treat-none” strategies across threshold probabilities between approximately 0.18 and 0.42 (**Supplementary Figure S3**). Similarly, the depression model showed clear net benefit across thresholds between 0.15 and 0.45 (**Supplementary Figure S4**). These findings indicate that applying the models to guide early psychological screening or nursing interventions provides superior clinical value compared with usual care.

## Discussion

In this large-scale retrospective study involving 1,420 hospitalized patients with chronic kidney disease (CKD), we developed and internally validated two nursing-oriented predictive models to estimate the risk of anxiety and depression using only routine inpatient data. Our findings revealed that approximately one-third of CKD inpatients exhibited symptoms of anxiety (33.2%) and depression (35.8%) upon admission, emphasizing the substantial and often underrecognized burden of psychological distress in this population. The models demonstrated excellent discriminative ability, with AUCs of 0.830 and 0.829 for anxiety and depression, respectively, and showed good calibration following 1,000 bootstrap replications. Given that all predictors and outcomes were measured at admission, this work should be interpreted as an early-stage model-development study using cross-sectional data with internal bootstrap validation rather than a fully deployable predictive instrument. Importantly, these models were constructed using variables readily available through routine nursing assessments, positioning them as practical, scalable tools for early psychological risk identification in nephrology wards. These findings reinforce the premise that nursing-oriented features are not merely supplementary but offer substantive prognostic value. Because they reflect continuous bedside observation rather than static demographic information, they enable a level of psychological risk detection that traditional population-based models cannot achieve. Unlike population-based models that predominantly rely on sociodemographic variables or laboratory biomarkers, our inclusion of nurse-observed factors such as sleep disturbance and in-hospital family support provides a more dynamic assessment of patients’ psychosocial burden, capturing aspects of emotional risk that typically go undetected in epidemiologic datasets. Although internal bootstrap validation supported the stability of the models, the lack of a separate validation cohort remains a limitation, and external validation in independent CKD inpatient populations is essential to determine generalizability. We also acknowledge that stepwise regression has well-recognized limitations; therefore, we incorporated LASSO penalization before multivariable modeling, which substantially reduces model optimism and enhances stability beyond what bootstrapping alone can provide.

Consistent with existing literature, our study identified younger age, female sex, low income, sleep disturbance, diabetes mellitus, hypoalbuminemia, and frequent hospitalizations as significant predictors of anxiety and depression in CKD patients (14, 15). Notably, the absence of family accompaniment emerged as an independent risk factor in both models, underscoring the psychosocial vulnerability of patients lacking in-hospital social support. This finding aligns with theories of social buffering and reinforces previous evidence that family presence during hospitalization mitigates emotional stress and improves clinical outcomes (16, 17). Sleep disturbance, captured through nurse observations, was among the strongest predictors of psychological distress. This variable likely reflects both poor sleep hygiene during hospitalization and underlying mood dysregulation, a bidirectional relationship that has been extensively documented in patients with chronic illness (18). Given its standardized documentation and high data completeness, we prioritized sleep disturbance as the sole symptom-level nursing indicator in the final models, while relying on comorbidities and core laboratory indices (e.g., diabetes mellitus, anemia, hypoalbuminemia) to represent the broader medical context.

Our models add to a growing body of literature by prioritizing nursing-accessible variables in psychological risk prediction—a design choice that has significant implications for real-world deployment. Prior predictive efforts have largely focused on outpatient or dialysis populations and frequently relied on complex, non-routine indicators—such as comprehensive inflammatory biomarker profiles (e.g., IL−6, TNF−α, CRP) and nutritional–inflammatory status measures—that require specialized laboratory testing rather than bedside nursing assessments (19). Additionally, some studies have incorporated systemic immune-inflammation indices (SII) derived from full blood counts to predict depression in dialysis-dependent patients, further illustrating the reliance on advanced, non-routine biomarker data for psychological risk modeling in CKD populations (20). In contrast, our models can be operationalized without additional diagnostic costs or external referrals, supporting a more integrated, task-shifted approach to inpatient mental health care. Furthermore, the use of visual nomograms enhances clinical interpretability and may facilitate embedding the model into electronic health record (EHR) systems, where automated alerts can prompt early referral to psychiatric consultation or the implementation of tiered nursing interventions. In addition, the accompanying web-based calculator translates the models into a simple point-of-care tool that can be used by bedside nurses without manual score conversion. This aligns with global efforts to strengthen mental health integration within chronic disease care, particularly in resource-constrained settings (21).

While we developed separate models for anxiety and depression, it is important to acknowledge the substantial clinical and biological overlap between these conditions. Previous studies have demonstrated that anxiety and depression frequently co-occur in patients with CKD, likely sharing underlying mechanisms such as systemic inflammation, hypothalamic–pituitary–adrenal (HPA) axis dysregulation, and neuroimmune alterations. For instance, a 2023 cohort study found elevated inflammatory cytokines and cortisol levels in CKD patients with coexisting anxiety and depressive symptoms, supporting inflammation–HPA axis interplay in mood dysregulation (22, 23) .The decision to model these outcomes separately was guided by their distinct clinical manifestations and potentially divergent intervention pathways. However, future research could explore the feasibility of developing unified or multilabel prediction frameworks that account for comorbidity patterns and offer enhanced screening efficiency.

In interpreting these findings, several plausible mechanisms warrant consideration. For instance, hypoalbuminemia—which was independently associated with both anxiety and depression—may serve as a surrogate for systemic inflammation and poor nutritional status. Emerging evidence in CKD shows that low serum albumin consistently correlates with elevated inflammatory markers and malnutrition−inflammation syndrome, both of which have been linked to mood disorders in this population (24, 25). Similarly, the association between frequent hospitalizations and psychological distress may reflect cumulative exposure to illness uncertainty, fragmented care, or financial stressors. Interestingly, dialysis status was an independent predictor of depression but not anxiety, suggesting that the chronic existential and lifestyle burden of renal replacement therapy may have a more pronounced influence on depressive symptomatology than acute hospital-related stress. These observations suggest the value of tailoring psychosocial interventions according to both treatment modality and individual patient trajectories.

Our models also raise important considerations for clinical implementation. While strong predictive performance is encouraging, the real-world utility of these tools depends on their integration into nursing workflows and their ability to inform actionable care pathways. For example, threshold scores could trigger structured psychological support protocols, including brief cognitive-behavioral interventions, family-centered counseling, or referrals to psychiatric care. Moreover, the cost-effectiveness of such risk-guided interventions should be explored in future prospective studies, particularly given the known associations between psychological distress, treatment nonadherence, and adverse renal outcomes.

This study has several strengths, including the use of a large and well-characterized inpatient cohort, reliance on validated psychological instruments (GAD-7, PHQ-9), and rigorous internal validation procedures. The focus on modifiable and nursing-accessible predictors enhances the model’s feasibility and potential for broad adoption. By integrating variables that arise directly from routine nursing assessments, our models extend beyond the limitations of population-level prediction tools and demonstrate how nursing data can be transformed into actionable insights for tailored psychological interventions. Despite the strengths of our study, several limitations must be acknowledged. First, the retrospective design inherently limits causal inference and is subject to information bias due to reliance on routinely documented electronic health records (EHRs). Key psychological and behavioral factors such as pain intensity, use of corticosteroids or sedative medications, cognitive impairment, and coping style were not systematically captured in structured EHR fields and therefore could not be incorporated into the models, leaving the possibility of residual confounding. Accordingly, the identified predictors should be viewed as markers of elevated psychological risk rather than definitive causal determinants, and future prospective studies incorporating detailed assessments of pain, medication exposure, cognitive function, and coping strategies are needed to clarify underlying mechanisms. Second, while anxiety and depression were assessed using the GAD-7 and PHQ-9—validated instruments in Chinese populations—these tools are screening measures and not substitutes for structured psychiatric diagnostic interviews. Accordingly, our models estimate the risk of elevated anxiety and depressive symptoms (psychological distress) rather than formal psychiatric disorders, and the reported prevalence reflects screening-positive cases based on symptom scales. Moreover, the possibility of response bias exists as patients completed these assessments under nurse supervision, which may have influenced reporting due to social desirability effects, particularly among older adults and individuals with lower educational attainment. Third, our model was developed using data from a single tertiary center in Southwest China, potentially limiting generalizability to other healthcare systems, ethnic groups, or sociocultural settings. Furthermore, while bootstrap validation reduces optimism bias, it does not replace validation using an independent dataset; therefore, multicenter external validation is warranted. The model’s predictive performance across CKD subgroups—such as dialysis versus non-dialysis patients, different CKD stages, or first-time versus frequent hospitalizations—was not separately evaluated, and external validation in diverse populations is necessary. Fourth, the study did not compare our nursing-oriented models against existing risk stratification tools developed for outpatient or dialysis populations, which limits the assessment of their relative incremental utility. Fifth, although our models offer practical bedside applications, they have yet to be tested in clinical practice to determine whether their use improves patient outcomes through targeted psychological interventions. Finally, selection bias may have occurred, as only patients who completed psychological assessments within 48 hours of admission were included. This may underrepresent individuals with communication barriers, cognitive dysfunction, or lack of caregiver support—ironically, those most vulnerable to psychological distress. The absence of temporal separation between predictors and outcomes, together with the lack of external validation, indicates that the models should be viewed as internally validated, cross-sectional risk-prediction tools requiring future prospective and multicenter external validation.

In conclusion, our study highlights the high prevalence and multifactorial nature of anxiety and depression among hospitalized CKD patients and introduces two internally validated predictive models grounded in routine nursing assessments. These models offer a novel, clinically pragmatic approach to early psychological risk stratification in nephrology care. Their potential integration into EHR systems and alignment with nurse-led mental health interventions represent important avenues for improving psychological outcomes in CKD populations. Future prospective validation, implementation studies, and impact evaluations are warranted to optimize their clinical utility and sustainability.

## Data Availability

The original contributions presented in the study are included in the article/**Supplementary Material**. Further inquiries can be directed to the corresponding author.

## Supplementary material

Supplementary Figure 1 LASSO coefficient profiles and tuning parameter (λ) selection for the anxiety prediction model. **(A)** LASSO coefficient profiles of 19 candidate predictors for anxiety across a sequence of log(λ) values. Each colored line represents the trajectory of a predictor’s coefficient as the penalty increases. Coefficients shrink progressively toward zero, and eight variables remain non-zero near the optimal λ, indicating their stability and contribution to the final model. **(B)** Tenfold cross-validation curve used to determine the optimal λ value based on minimum binomial deviance. Dots represent mean cross-validated deviance, and grey bars indicate standard errors. The left dashed line denotes the λ with minimum deviance (λ_min), while the right dashed line denotes the 1-SE criterion (λ_1SE). At λ_min, eight non-zero coefficients were selected, corresponding exactly to the independent predictors identified in multivariate logistic regression (age ≥60, female sex, low income, ≥2 hospitalizations, hypoalbuminemia, sleep disturbance, lack of family accompaniment, and diabetes mellitus).

Supplementary Figure 2 LASSO coefficient profiles and tuning parameter (λ) selection for the depression prediction model. **(A)** LASSO coefficient profiles of candidate predictors for depression across a sequence of log(λ) values. Each colored line represents the evolution of a predictor’s coefficient as the regularization penalty increases. As λ increases, coefficients shrink toward zero, and nine variables remain non-zero near the optimal λ, indicating their stability and contribution to the penalized regression model. **(B)** Tenfold cross-validation curve for selection of the optimal λ value based on minimum binomial deviance. Blue dots represent mean cross-validated deviance, with grey error bars indicating standard errors. The left dashed line denotes λ_min (minimum deviance), while the right dashed line represents λ_1SE (one-standard-error rule). At λ_min, nine non-zero coefficients were retained, corresponding to predictors selected for model construction.

Supplementary Figure 3 Decision-curve analysis for the anxiety risk-prediction model. Decision-curve analysis (DCA) was performed to evaluate the clinical utility of the anxiety prediction model across a range of threshold probabilities. The model demonstrated a consistently higher net benefit than the “treat-all” and “treat-none” strategies within the threshold range of approximately 0.18–0.42. Individual predictor curves (e.g., age ≥60 years, female sex, low income, ≥2 hospitalizations, low albumin, sleep disturbance, no family accompaniment, diabetes) are shown for comparison and yield substantially lower net benefit. The results indicate that using the full multivariable model provides superior clinical value over single-factor decision strategies when guiding early psychological screening or nursing interventions for anxiety among CKD inpatients.

Supplementary Figure 4 Decision-curve analysis for the depression risk-prediction model. Decision-curve analysis (DCA) was used to assess the net clinical benefit of the depression prediction model across a continuum of threshold probabilities. The model offered greater net benefit than the “treat-all” and “treat-none” approaches between threshold probabilities of approximately 0.15–0.45. Individual predictor curves (such as age ≥60 years, female sex, low education, low income, dialysis, ≥2 hospitalizations, low albumin, and sleep disturbance) showed inferior net benefit compared with the full model. These findings indicate that the multivariable model provides more advantageous decision-making support than single-predictor strategies and may assist nurses in identifying hospitalized CKD patients at elevated risk of depressive symptoms.

## References

[1] Kovesdy CP . Epidemiology of chronic kidney disease: an update 2022. Kidney Int Suppl (2011). (2022) 12:7–11. doi: 10.1016/j.kisu.2021.11.003, PMID: 35529086 PMC9073222

[2] Chilcot J Pearce CJ Hall N Rehman Z Norton S Griffiths S . Depression and anxiety in people with kidney disease: understanding symptom variability, patient experience and preferences for mental health support. J Nephrol. (2025) 38:675–86. doi: 10.1007/s40620-024-02194-1, PMID: 39799543 PMC11961520

[3] Yang H Qi L Pei D . Effect of psychosocial interventions for depression in adults with chronic kidney disease: a systematic review and meta-analysis. BMC Nephrol. (2024) 25:17. doi: 10.1186/s12882-023-03447-0, PMID: 38200465 PMC10782786

[4] Ford DM Budworth L Lawton R Teale EA O'Connor DB . In-hospital stress and patient outcomes: A systematic review and meta-analysis. PloS One. (2023) 18:e0282789. doi: 10.1371/journal.pone.0282789, PMID: 36893099 PMC9997980

[5] Barbosa GM Weber A Garcia APRF Toledo VP . Experience of hospitalization of the family with children and adolescents in psychological distress. Rev Esc Enferm USP. (2023) 57:e20220457. doi: 10.1590/1980-220X-REEUSP-2022-0457en, PMID: 37930233 PMC10615362

[6] Arooj H Aman M Hashmi MU Nasir Z Zahid M Abbas J . The impact of nurse-led care in chronic kidney disease management: a systematic review and meta-analysis. BMC Nurs. (2025) 24:188. doi: 10.1186/s12912-025-02829-z, PMID: 39966917 PMC11837475

[7] Yang M Tang X Fang Y . Analysis of risk factors for depression in peritoneal dialysis patients and establishment of a risk nomogram model. Clinics (Sao Paulo). (2025) 80:100600. doi: 10.1016/j.clinsp.2025.100600, PMID: 39951876 PMC11874718

[8] Chilcot J Pearce CJ Hall N Busby AD Hawkins J Vraitch B . The identification and management of depression in UK Kidney Care: Results from the Mood Maps Study. J Ren Care. (2024) 50:297–306. doi: 10.1111/jorc.12489, PMID: 38341770

[9] AlShammari OA AlFadil SO AlShabibi A Mohamed H Alomi M Almatham K . Prevalence of anxiety and depression among end-stage kidney disease patients on dialysis: A cross-sectional multiple-centre study in Riyadh, Saudi Arabia. J Family Med Prim Care. (2024) 13:4406–12. doi: 10.4103/jfmpc.jfmpc_355_24, PMID: 39629409 PMC11610806

[10] Ma J Ma DW . Advancements in the application of precision nursing model on hemodialysis for diabetic nephropathy: A review. Med (Baltimore). (2024) 103:e40952. doi: 10.1097/MD.0000000000040952, PMID: 39705468 PMC11666179

[11] Yan Q Liu G Wang R Li D Wang D . Development and validation of a nomogram for predicting depression risk in patients with chronic kidney disease based on NHANES 2005-2018. J Health Popul Nutr. (2025) 44:136. doi: 10.1186/s41043-025-00890-7, PMID: 40281636 PMC12023547

[12] Zha B Cai A Yu H Wang Z . Development and validation of a predictive model for depression in patients with advanced stage of cardiovascular-kidney-metabolic syndrome. J Affect Disord. (2025) 383:32–40. doi: 10.1016/j.jad.2025.04.139, PMID: 40280435

[13] Zhou X Zhu F . Development and validation of a nomogram model for accurately predicting depression in maintenance hemodialysis patients: A multicenter cross-sectional study in China. Risk Manag Healthc Policy. (2024), 17:2111–2123. doi: 10.2147/RMHP.S456499, PMID: 39246589 PMC11380485

[14] Kim S Jeon J Lee YJ Jang HR Joo EY Huh W . Depression is a main determinant of health-related quality of life in patients with diabetic kidney disease. Sci Rep. (2022) 12:12159. doi: 10.1038/s41598-022-15906-z, PMID: 35842489 PMC9288542

[15] Huang J Mao Y Zhao X Liu Q Zheng T . Association of anxiety, depression symptoms and sleep quality with chronic kidney disease among older Chinese. Med (Baltimore). (2023) 102:e35812. doi: 10.1097/MD.0000000000035812, PMID: 37904348 PMC10615427

[16] Shulyaev K Spielberg Y Gur-Yaish N Zisberg A . Family support during hospitalization buffers depressive symptoms among independent older adults. Clin Gerontol. (2024) 47:341–51. doi: 10.1080/07317115.2023.2236097, PMID: 37493087

[17] Duong J Wang G Lean G Slobod D Goldfarb M . Family-centered interventions and patient outcomes in the adult intensive care unit: A systematic review of randomized controlled trials. J Crit Care. (2024) 83:154829. doi: 10.1016/j.jcrc.2024.154829, PMID: 38759579

[18] Yasugaki S Okamura H Kaneko A Hayashi Y . Bidirectional relationship between sleep and depression. Neurosci Res. (2025) 211:57–64. doi: 10.1016/j.neures.2023.04.006, PMID: 37116584

[19] Graterol Torres F Molina M Soler-Majoral J Romero-González G Rodríguez Chitiva N Troya-Saborido M . Evolving concepts on inflammatory biomarkers and malnutrition in chronic kidney disease. Nutrients. (2022) 14:4297. doi: 10.3390/nu14204297, PMID: 36296981 PMC9611115

[20] Han XX Zhang HY Kong JW Liu YX Zhang KR Ren WY . Systemic immune inflammation index is a valuable marker for predicting hemodialysis patients with depression: a cross-sectional study. Front Psychiatry. (2024) 15:1423200. doi: 10.3389/fpsyt.2024.1423200, PMID: 39161547 PMC11331312

[21] Buchanan GJR Berge JM,F Piehler T . Integrated behavioral health implementation and chronic disease management inequities: an exploratory study of statewide data. BMC Prim Care. (2024) 25:302. doi: 10.1186/s12875-024-02483-5, PMID: 39143518 PMC11323651

[22] Qin C Wu Y Zou Y Zhao Y Kang D Liu F . Associations between depressive and anxiety symptoms and incident kidney failure in patients with diabetic nephropathy. BMC Nephrol. (2025) 26:54. doi: 10.1186/s12882-025-03983-x, PMID: 39905282 PMC11796097

[23] Sagmeister MS Harper L Hardy RS . Cortisol excess in chronic kidney disease - A review of changes and impact on mortality. Front Endocrinol (Lausanne). (2023) 13:1075809. doi: 10.3389/fendo.2022.1075809, PMID: 36733794 PMC9886668

[24] Wang CH Jiang MH Ma JM Yuan MC Liao L Duan HZ . Identification of independent risk factors for hypoalbuminemia in patients with CKD stages 3 and 4: the construction of a nomogram. Front Nutr. (2024) 11:1453240. doi: 10.3389/fnut.2024.1453240, PMID: 39545043 PMC11562854

[25] Park IH Ko NG Jin M Lee YJ . Lower prognostic nutritional index is associated with a greater decline in long-term kidney function in general population. Nutr J. (2024) 23:146. doi: 10.1186/s12937-024-01047-8, PMID: 39567944 PMC11580526

