# Supplementary material for: Development and validation of nursing-oriented risk prediction models for anxiety and depression in hospitalized patients with chronic kidney disease: a retrospective cross-sectional study in Southwest China
Source: Front Psychiatry. 2026 Jan 15;16:1683467. doi: 10.3389/fpsyt.2025.1683467 (PMC12852360; doi:10.3389/fpsyt.2025.1683467)
Supplement: Supplementary file 2 [file Table1.docx]

**Supplementary Table 1.** LASSO-selected variables and corresponding coefficients at λ_min for the anxiety prediction model

| **Variable** | **Coefficient at λ_min** |
| --- | --- |
| **age60** (≥60 years) | −0.3245 |
| **female** | 0.2469 |
| **low_income** (<3,000 RMB/month) | 0.5238 |
| **hosp_ge2** (≥2 hospitalizations in past year) | 0.4337 |
| **alb_low** (<35 g/L) | 0.4449 |
| **sleep_disturbance** | 0.8489 |
| **no_family_accompaniment** | 0.5407 |
| **diabetes_mellitus** | 0.2102 |

**Note:** LASSO regression was performed using a 10-fold cross-validation framework. At the optimal λ_min based on minimum binomial deviance, eight variables exhibited non-zero coefficients, indicating their contribution to the penalized model. These variables exactly matched the independent predictors identified in the multivariate logistic regression model (Table 3), supporting the robustness and consistency of variable selection. Negative coefficients represent protective effects, whereas positive coefficients indicate higher anxiety risk. The intercept at λ_min was −1.8022.

**Supplementary Table 2.** LASSO-selected variables and corresponding coefficients at λ_min for the depression prediction model

| **Variable** | **Coefficient at λ_min** |
| --- | --- |
| **age60** (≥60 years) | −0.1369 |
| **female** | 0.2707 |
| **edu_low** (≤ middle school) | 0.3893 |
| **low_income** (<3,000 RMB/month) | 0.4294 |
| **dialysis** | 0.1983 |
| **hosp_ge2** (≥2 hospitalizations in past year) | 0.7212 |
| **alb_low** (<35 g/L) | 0.2829 |
| **sleep_disturbance** | 0.8270 |
| **no_family_accompaniment** | 0.6804 |

**Note:** LASSO regression using 10-fold cross-validation identified nine non-zero coefficients at the optimal λ_min. These variables represent the most stable predictors contributing to the penalized logistic regression model for depression. The direction and magnitude of coefficients reflect the associated risk: positive values indicate increased depression risk, whereas negative values represent protective effects. The intercept at λ_min was −1.8490. LASSO selection was consistent with variables identified as significant predictors in multivariate logistic regression, supporting the robustness of variable selection.
