# Supplementary figures and images for: Development and validation of nursing-oriented risk prediction models for anxiety and depression in hospitalized patients with chronic kidney disease: a retrospective cross-sectional study in Southwest China
Source: Front Psychiatry. 2026 Jan 15;16:1683467. doi: 10.3389/fpsyt.2025.1683467 (PMC12852360; doi:10.3389/fpsyt.2025.1683467)

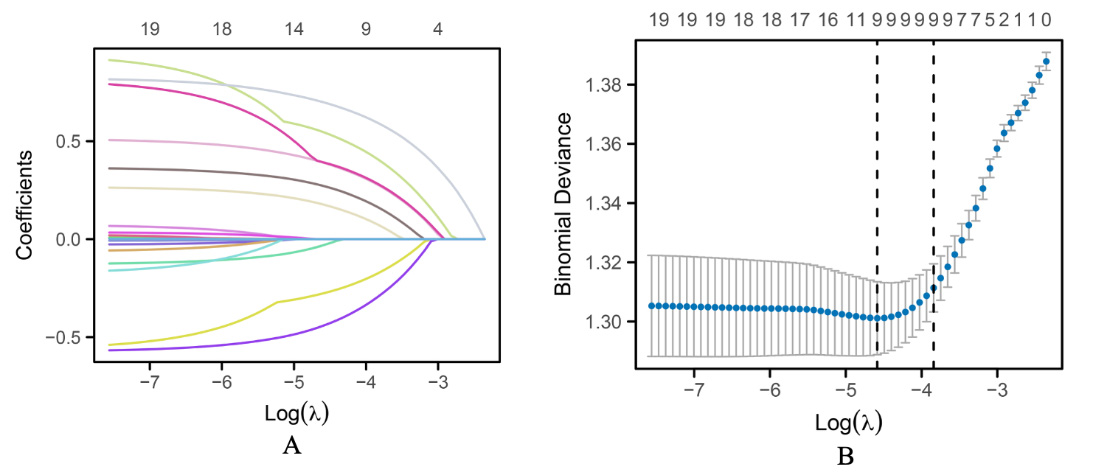

Supplement: Supplementary Figure 1 — LASSO coefficient profiles and tuning parameter (λ) selection for the anxiety prediction model. (A) LASSO coefficient profiles of 19 candidate predictors for anxiety across a sequence of log(λ) values. Each colored line represents the trajectory of a predictor’s coefficient as the penalty increases. Coefficients shrink progressively toward zero, and eight variables remain non-zero near the optimal λ, indicating their stability and contribution to the final model. (B) Tenfold cross-validation curve used to determine the optimal λ value based on minimum binomial deviance. Dots represent mean cross-validated deviance, and grey bars indicate standard errors. The left dashed line denotes the λ with minimum deviance (λ_min), while the right dashed line denotes the 1-SE criterion (λ_1SE). At λ_min, eight non-zero coefficients were selected, corresponding exactly to the independent predictors identified in multivariate logistic regression (age ≥60, female sex, low income, ≥2 hospitalizations, hypoalbuminemia, sleep disturbance, lack of family accompaniment, and diabetes mellitus). [file Image1.jpeg]

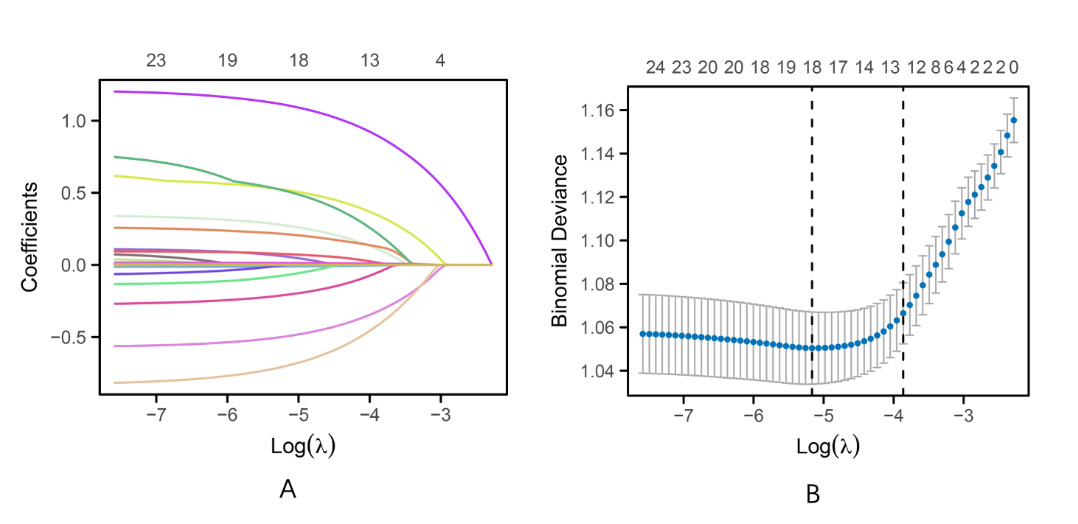

Supplement: Supplementary Figure 2 — LASSO coefficient profiles and tuning parameter (λ) selection for the depression prediction model. (A) LASSO coefficient profiles of candidate predictors for depression across a sequence of log(λ) values. Each colored line represents the evolution of a predictor’s coefficient as the regularization penalty increases. As λ increases, coefficients shrink toward zero, and nine variables remain non-zero near the optimal λ, indicating their stability and contribution to the penalized regression model. (B) Tenfold cross-validation curve for selection of the optimal λ value based on minimum binomial deviance. Blue dots represent mean cross-validated deviance, with grey error bars indicating standard errors. The left dashed line denotes λ_min (minimum deviance), while the right dashed line represents λ_1SE (one-standard-error rule). At λ_min, nine non-zero coefficients were retained, corresponding to predictors selected for model construction. [file Image2.jpeg]

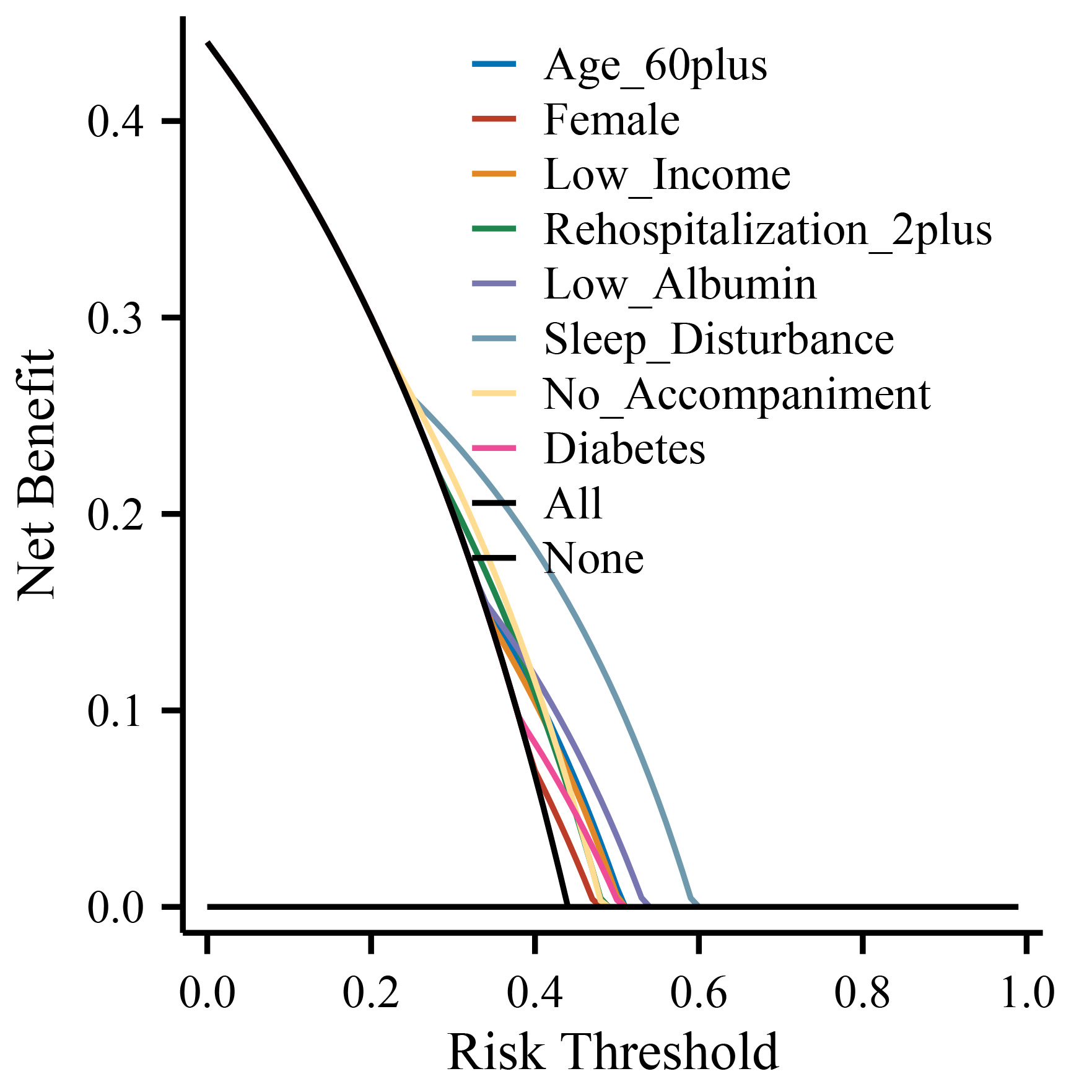

Supplement: Supplementary Figure 3 — Decision-curve analysis for the anxiety risk-prediction model. Decision-curve analysis (DCA) was performed to evaluate the clinical utility of the anxiety prediction model across a range of threshold probabilities. The model demonstrated a consistently higher net benefit than the “treat-all” and “treat-none” strategies within the threshold range of approximately 0.18–0.42. Individual predictor curves (e.g., age ≥60 years, female sex, low income, ≥2 hospitalizations, low albumin, sleep disturbance, no family accompaniment, diabetes) are shown for comparison and yield substantially lower net benefit. The results indicate that using the full multivariable model provides superior clinical value over single-factor decision strategies when guiding early psychological screening or nursing interventions for anxiety among CKD inpatients. [file Image3.jpeg]

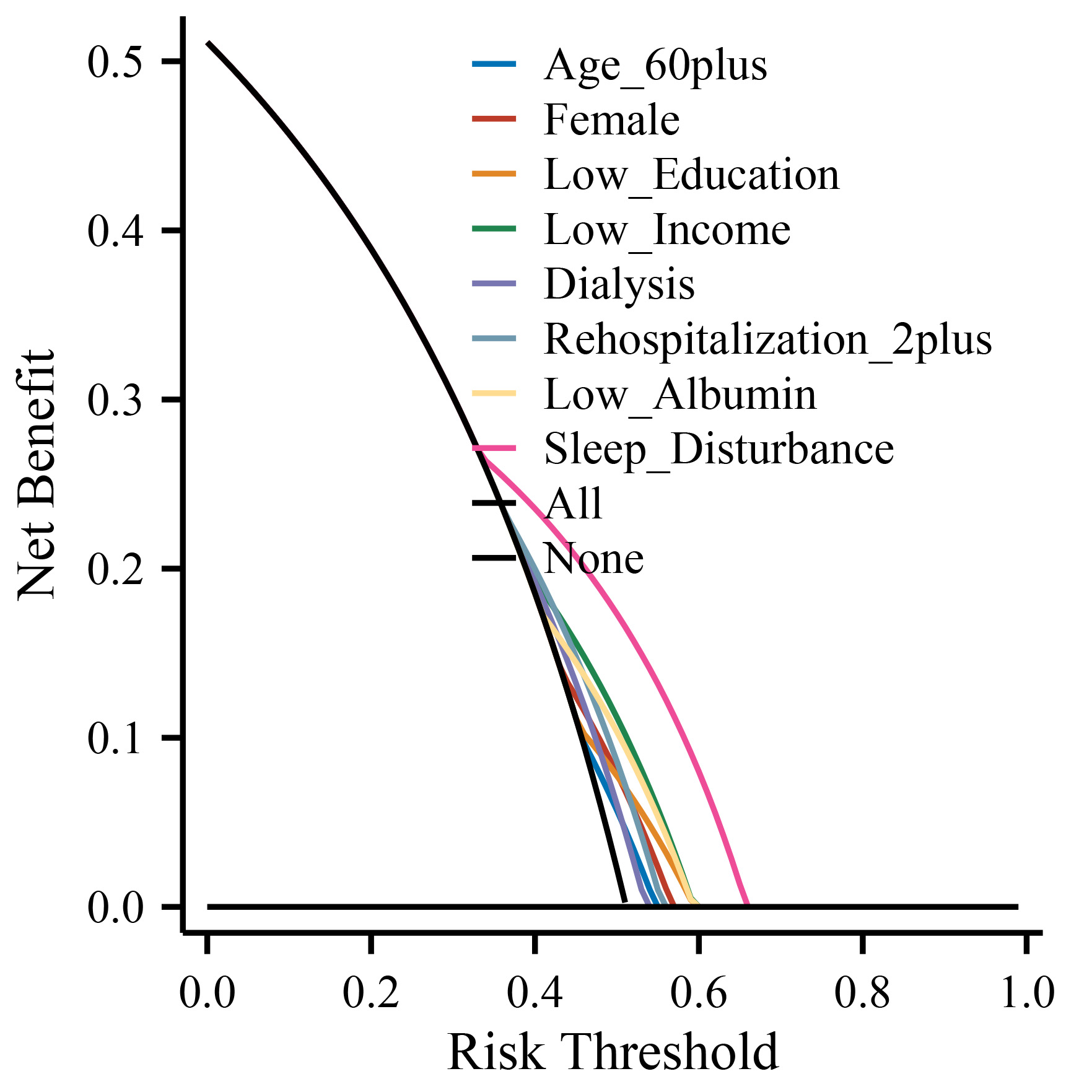

Supplement: Supplementary Figure 4 — Decision-curve analysis for the depression risk-prediction model. Decision-curve analysis (DCA) was used to assess the net clinical benefit of the depression prediction model across a continuum of threshold probabilities. The model offered greater net benefit than the “treat-all” and “treat-none” approaches between threshold probabilities of approximately 0.15–0.45. Individual predictor curves (such as age ≥60 years, female sex, low education, low income, dialysis, ≥2 hospitalizations, low albumin, and sleep disturbance) showed inferior net benefit compared with the full model. These findings indicate that the multivariable model provides more advantageous decision-making support than single-predictor strategies and may assist nurses in identifying hospitalized CKD patients at elevated risk of depressive symptoms. [file Image4.jpeg]
